# Using intrinsic properties of quantum dots to provide additional security when uniquely identifying devices

**DOI:** 10.1038/s41598-022-20596-8

**Published:** 2022-10-08

**Authors:** Matthew J. Fong, Christopher S. Woodhead, Nema M. Abdelazim, Daniel C. Abreu, Angelo Lamantia, Elliott M. Ball, Kieran Longmate, David Howarth, Benjamin J. Robinson, Phillip Speed, Robert J. Young

**Affiliations:** 1 Physics Department, Lancaster University, Bailrigg, LA1 4YB UK; 2 Quantum Base Ltd, Department of Physics, Lancaster University, Bailrigg, LA1 4YB UK; 3 Present Address: School of Electronics and Computer Science, University of Southampton, Southampton, SO17 1BJ UK

**Keywords:** Quantum dots, Nanophotonics and plasmonics, Imaging techniques

## Abstract

Unique identification of optical devices is important for anti-counterfeiting. Physical unclonable functions (PUFs), which use random physical characteristics for authentication, are advantageous over existing optical solutions, such as holograms, due to the inherent asymmetry in their fabrication and reproduction complexity. However, whilst unique, PUFs are potentially vulnerable to replication and simulation. Here we introduce an additional benefit of a small modification to an established model of nanoparticle PUFs by using a second measurement parameter to verify their authenticity. A randomly deposited array of quantum dots is encapsulated in a transparent polymer, forming a tag. Photoluminescence is measured as a function of excitation power to assess uniqueness as well as the intrinsic nonlinear response of the quantum material. This captures a fingerprint, which is non-trivial to clone or simulate. To demonstrate this concept practically, we show that these tags can be read using an unmodified smartphone, with its built-in flash for excitation. This development over constellation-style optical PUFs paves the way for more secure, facile authentication of devices without requiring complex fabrication or characterisation techniques.

## Introduction

There is a continuous arms race to create increasingly more complex optical tags for the secure verification and authentication of devices. Manufacturers have developed more convoluted fabrication methods to create devices that are increasingly difficult to replicate by a copycat. However, when security is underpinned by manufacturing complexity and the processes used are known to all parties, there is a level playing field between genuine manufacturers and counterfeiters. This symmetry in fabrication, where it is as complex to fabricate a security device for a nefarious party as it is for the original manufacturer, lowers the bar to replicate security devices. For example, the intrinsic security of a hologram arises from the complexity and cost of fabrication, so as technology improves and costs reduce, it becomes easier to replicate, creating a game of cat and mouse between manufacturers and counterfeiters. Additionally, these devices use assumed knowledge on the behalf of the consumer, that they can tell a genuine device from a counterfeit, which can cause a degree of human error in verification.

To resolve these issues, there has been increasing interest in optical physical unclonable functions (PUFs). A PUF is a system that can generate a unique set of responses, to a given challenge (known as challenge-response pairs), using intrinsic properties from the device. A key figure of merit of the PUF is the asymmetric fabrication and measurement process. Unlike symmetrical security devices, PUFs use the intrinsic unpredictability and irreproducibility of the fabrication method as the source of the security, creating unique devices each and every time. The asymmetrical fabrication ensures it is trivial to fabricate a unique and complex tag, but difficult, time-consuming and expensive to replicate. First demonstrated with the scattering of a laser through randomly distributed glass nanospheres to generate a unique pattern, this technique has evolved but remains fundamentally the same^1^.

To be scalable and accessible, a PUF needs not only to have a high entropy density with a large number of challenge-response pairs (CRPs), but it must be trivial for a user to authenticate^2^. A PUF with high entropy density and an asymmetry between fabrication and measurement, whilst theoretically powerful, is not practical in the real world if its properties cannot be measured and interpreted easily by a consumer. Quantum dots (QDs) and other nanoparticles have been widely discussed as potential PUFs due to their small size and low cost^3,4^. As optical emitters for PUFs, colloidal quantum dots are attractive candidates: ligands allow them to be suspended in many different solvents, inks or lacquers, which provides flexibility in the deposition process and solution concentration^5,6^, and precise control of their composition and size gives high tunability of their emission properties due to the highly sensitive confinement energy at this scale^7,8^.

Several optical PUFs have been proposed which rely on the random distribution of nanoparticles. As a result of their fabrication method, the spatial position of the nanoparticles is unpredictable and unique in their arrangement. Examples of nanoparticle PUFs (NP PUFs) include plasmonic nanoparticles which absorb and shift the wavelength of incident light^9,10^, the position of light-emitting nanowires^11^, or QDs randomly deposited on surfaces or embedded in 3D objects^12–15^. These PUFs rely on the random position of NPs, so to imitate them would require printing or simulation at the resolution of the verification device^16^, but often require more complex characterisation methods, which are less practical for real-world applications^17,18^.

In this work, we investigate the feasibility of adding a new dimension of security measurements to these constellation-style NP PUFs. We aim to demonstrate an improvement to this existing device type, by utilising the simple additional measurement of the QD response to deterministically increasing excitation intensity. Quantum dots exhibit a nonlinear emission in response to a linear increase in incident excitation power, which results from the quantum mechanics that dictates their behaviour. This can be used as a second dimension of security for verification of these devices, complementing their uses as fingerprints for secure authentication.

Emission from the radiative decay of neutral excitons confined in quantum emitters, such as quantum dots, saturates as the pump rate approaches the exciton’s radiative lifetime:

$${R}_{em}= \frac{{R}_{sat}}{\left(1+ \frac{{I}_{sat}}{{I}_{ex}}\right)}$$

where R_sat_ and R_em_ is the optical response rate at saturation point and emission, and I_sat_ and I_ex_ are the incident intensities at saturation and excitation^19^. This response makes a tag more difficult to replicate or simulate than a more conventional NP PUF because it is a difficult response to artificially recreate. At low excitation power, the primary emission mechanism for the sample arises from single exciton emission, but as the incident intensity increases, the emission from larger exciton states dominates, until eventually, it saturates at the point where the ground state of the QD is always occupied by an electron–hole pair^20^. We use this nonlinearity, in addition to the intensity-dependent response rates and switch-on points to establish an additional set of tests for verification of the authenticity of these devices.

Samples were fabricated with a simple, scalable technique using colloidal quantum dots electro sprayed onto a Si/SiO_2_ + Al surface. Details of this can be found in previous work but will be summarised here^21^. Colloidal InP/ZnS quantum dots (QDs) in a polar solution were ejected from a syringe with a fine needle, using a strong electrostatic force created by a large potential difference applied between the substrate and the needle. This creates a very fine aerosol from the end of the needle, separating the solution into many microscopic droplets of QD solution, ensuring the solvent evaporates before it reaches the surface, to aid a more uniform distribution of quantum dots. The dots were deposited onto a Si/SiO_2_ substrate, with a thin reflective layer of Al on its surface. As the Al layer has a coefficient of reflectivity close to 1, it reflects the majority of any unabsorbed light back towards the QDs, and light emitted by the QDs towards the CCD^22^.

**Figure 1:**
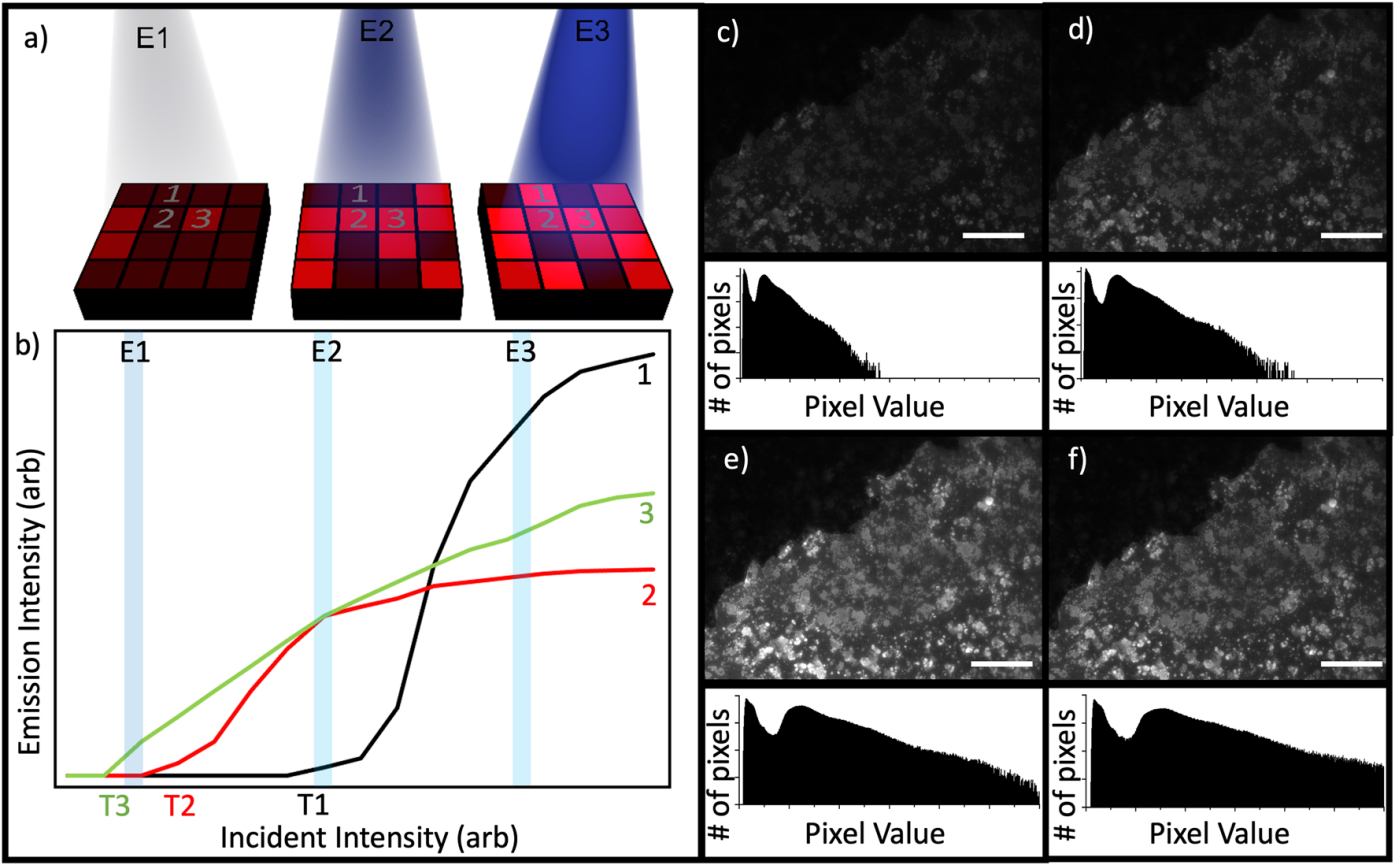
The concept of using the non-linear response of a quantum dot-based tag for authentication. (**a**) Illustrates a security tag’s surface, coated in quantum dots and divided into pixels for clarity. As the incident illumination increases (from left to right at 3 excitation powers, E1, E2, E3) the average amount of photoluminescence (shown in red) increases, but at different rates from region to region. T1, T2 and T3 show the switch-on intensities of different QD regions. (**b**) Sketches how the photoluminescence intensity from three example locations could vary differently with increasing excitation. (**c**)–(**f**) The top section of each panel shows photoluminescence from a thin film containing colloidal quantum dots at increasing excitation power, with pixel histograms underneath displaying the number of pixels recording each intensity value (0 to 65,355) at the different incident powers: 36.4 μW (**c**), 57.6 μW (**d**), 91.4 μW (**e**), and 145 μW (**f**). Scale bar represents 50 μm.

Figure 1a shows a schematic of a quantum dot sample, illuminated by three increasing excitation powers E1, E2 and E3. Due to the unpredictable deposition method of colloidal quantum dots, random clustering and changes in local density occur on the surface. The impact of different substrates and the random density of nanoparticles creates different switch-on regions of the sample. We define the switch-on intensity of the QDs as the intensity at which the CCD can detect the emission, above its noise threshold. Clusters of quantum dots have a lower switch-on threshold than regions with lower densities, creating another optical effect to verify the authenticity of the tags (Fig. 1b). Different particle sizes and densities of QDs on a surface will respond in various ways to the incident light, due to energy traps created in higher concentrations of dots^23^. Clusters are brighter, due to their higher density of quantum emitters, but if too densely clustered they begin to quench their emission^24,25^.

## Results

Figure 1c–f shows PL images and pixel-value histograms taken from a QD sample that was illuminated with increasing intensities, showing the variation in response. Two distinct peaks can be seen, the ratio and width of which do not remain constant with varying incident intensity. This nonuniform response across the sample is a characteristic pattern that can be used to confirm the authenticity of the tag. Both the position and the ratio of the two peaks with a variable incident power is a key additional layer of security.

**Figure 2:**
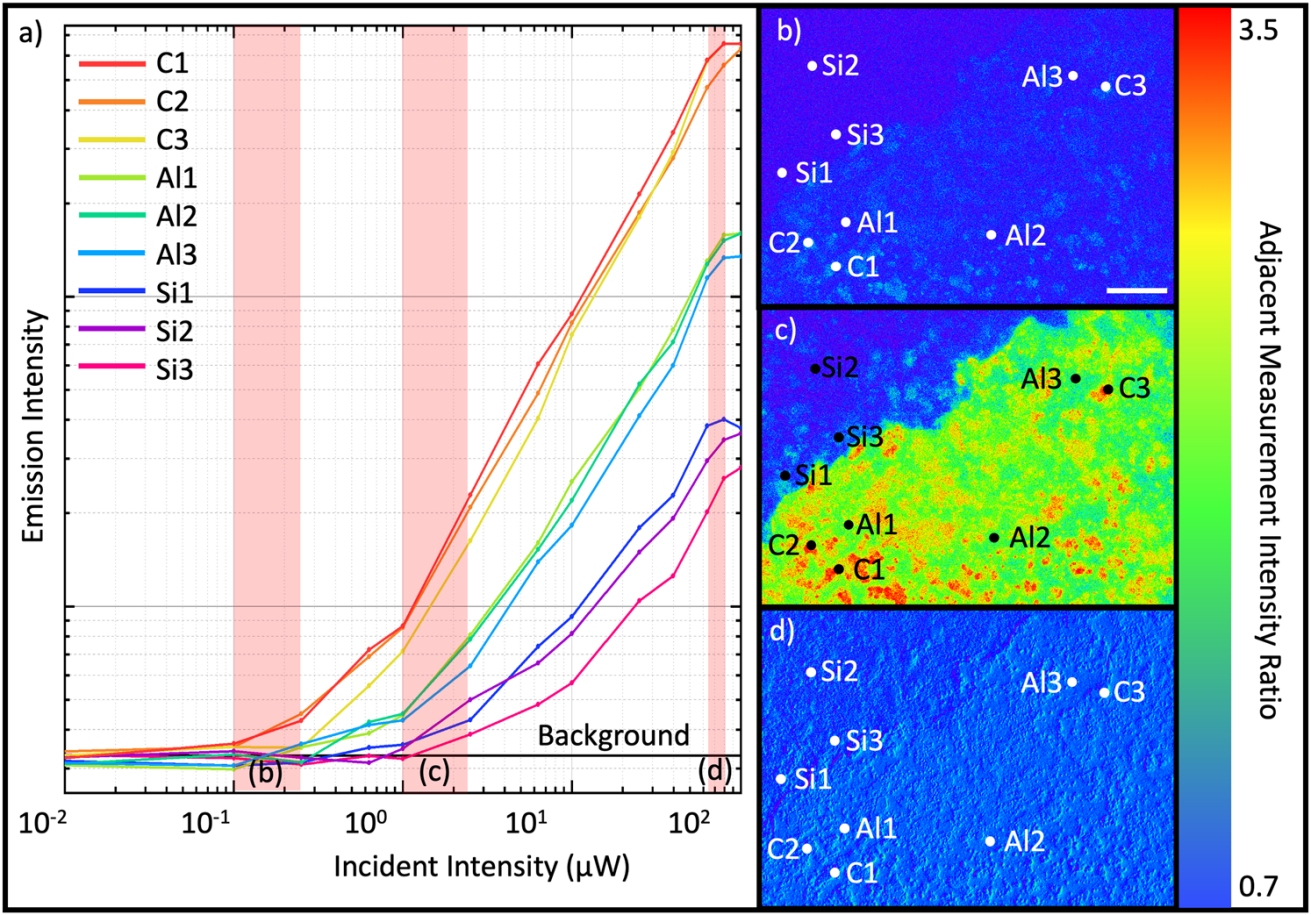
The non-linear optical response of a colloidal quantum dot film. (**a**) Log–log plot showing the emission intensity from a series of points chosen to represent 3 different region types on the sample: QDs on the Si substrate (labels Si1-3), QDs on the Al layer (Al1-3), and QD clusters on the surface (C1-3). (**b**)–(**d)** Show images created by taking the ratio of emission intensity maps recorded at adjacent excitation intensities. These are (**b**) 0.4/0.15 μW, (**c**) 3.6/1.4 μW and (**d**) 115/90 μW. Scale bar represents 50 μm. Z scale represents the relative difference in point-to-point variation on the sample surface.

PL measurements from three different types of region on the sample are shown in Fig. 2a: ‘background’ regions, where the dots were deposited at a low density on the bare substrate (labels beginning with ‘Si’), a low-density region, in which the dots are on the Al film (labels beginning with ‘Al’), and regions containing larger clusters of CQDs on the Al substrate (labels beginnings with ‘C’). A nonlinear increase in emission intensity with a linearly increasing excitation power can be seen, as well as variations in the switch-on intensity. Here, switch-on intensity is defined as the power at which the emission intensity measured by the CCD is 2 × the background level, which is the sample measured with no excitation intensity. Images showing the ratio of emission intensity and consecutive excitation powers are shown in Fig. 2b–d. These demonstrate the relative change in intensity between adjacent measurements of increasing incident intensity. Brighter areas on these maps indicate a greater relative increase. Different regions clearly have differing responses to the increasing incident intensity. The image in Fig. 2b was created by dividing the PL map captured at an illumination power of 0.40 μW by the one captured at 0.15 μW. The larger clusters on the Al substrate can be seen to switch on first, which is where the regions with the steepest gradient are mainly confined. These regions switch earlier than others on the sample, so the gradient remains constant at all other areas on the surface. Figure 2c is the result of dividing PL captured with an excitation power of 3.6 μW by the image captured at 1.4 μW, where it is clear the steepest gradient still comes from the clusters, whilst the Si background region remains very uniform. We can also see a uniform increase in the background level on the aluminium substrate. Figure 2d shows an image created by taking the ratio of the PL captured at 115 μW and 90 μW excitation powers, where the intensity increase is uniform across all areas of the sample as the measurement begins to saturate. The Z scale in Fig. 2b–d demonstrates the range of values for these divided images, demonstrating the much wider variation in emission range after the cluster switch-on, before saturation of the QDs.

**Figure 3:**
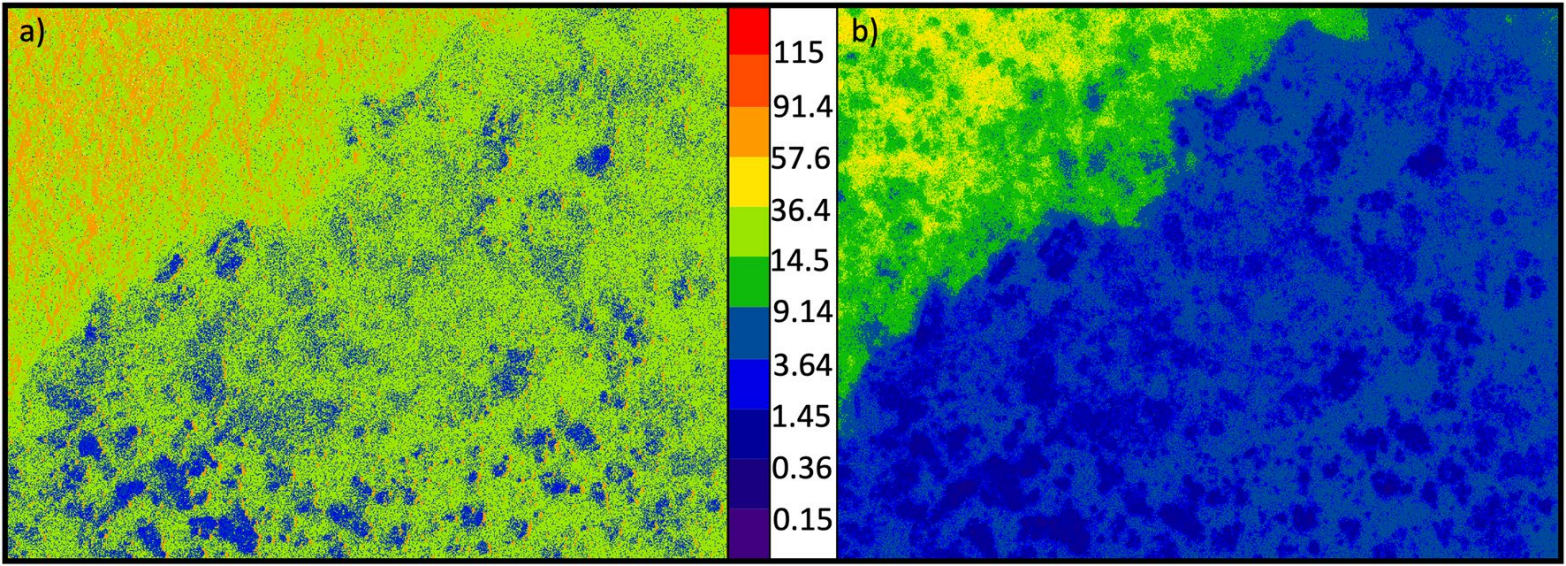
How the emission from different spatial regions responds to changes in incident power. (**a**) shows the excitation intensity difference at which there is the greatest relative increase in emission intensity. (**b**) shows the ‘switch-on’ intensity, i.e. the excitation power at which the emission intensity exceeds the background level. The colour scale indicates the incident power (μW).

To illustrate further how the regions containing clusters of QDs behave differently with excitation strength to the other regions, Fig. 3a shows the incident intensity at which each pixel has the greatest percentage increase. We see the regions which switch on at a lower excitation power are the largest clusters, and the rest of the sample has its greatest increase at a similar power, independent of the substrate. Figure 3b displays the ‘switch-on’ intensity of the samples, where the emission intensity become significant in comparison to the noise from the sensor. The colour scale shows incident power at which the emission intensity from the sample is greater than the background level, as determined by the measurement of the sample with no excitation power. It clearly demonstrates the switch-on of the largest clusters is at the lowest intensity, and areas with lower densities of QDs require a higher incident intensity to begin emitting. These patterns arise from both the substrate and random distribution of the QDs during the fabrication process. The large blue areas where the Al substrate is present shows the switch-on of the QDs here is at a much lower incident power than regions without Al.

In Fig. 3a, we observe the majority of the sample has the greatest relative increase between 14.5 and 36.4 μW (the light green areas), which extends over the whole sample, both on the Si/SiO_2_ substrate, and the Al coating. However, when we look instead for switch-on intensity, there is a clear definition around the edge of the Al in Fig. 3b, which is indicative that the Al significantly changes the switch-on intensity of the quantum dots but does not alter the peak gradient of emission rate. This is due to the Al film acting as a mirror and increasing the incident light absorbed by the QDs, reflecting any unabsorbed light in a useful direction, so the ‘switch-on’ point for the uniform distribution of QDs happens earlier, but the Al has no significant effect on the gradient of increase of emission intensity, which is related to the density of QDs on the surface. This is a factor that can be much more unpredictable during fabrication.

### Smartphone tag verification

**Figure 4:**
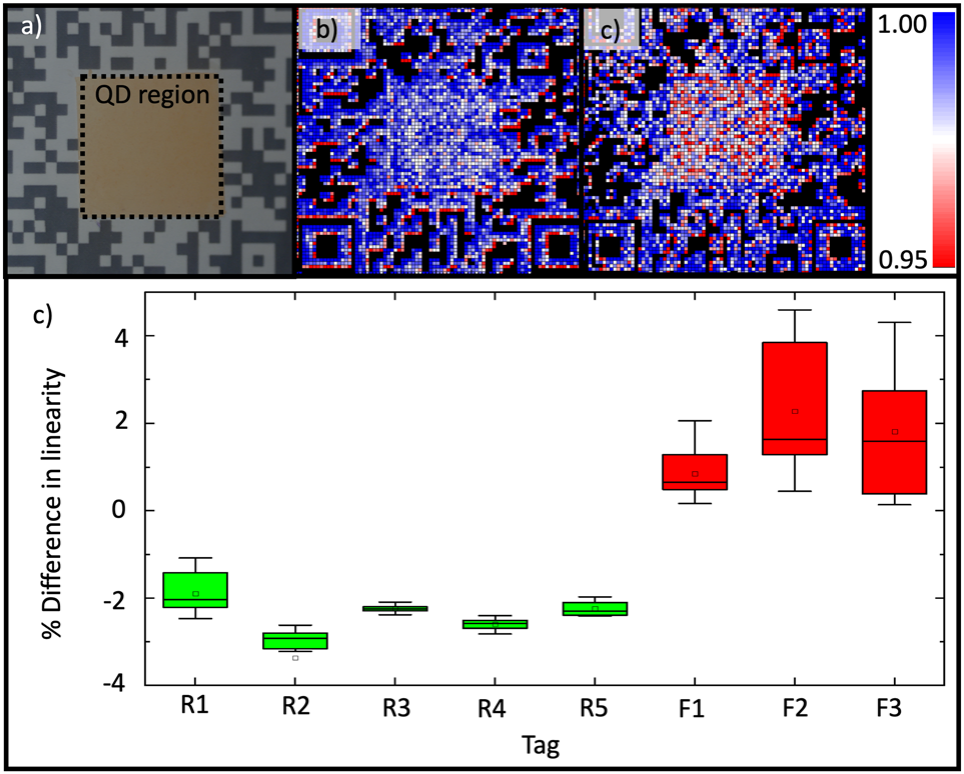
Measuring the non-linear response of a quantum dot-based tag using a smartphone’s camera and flash. (**a**) A smartphone image of a tag comprising a QR Code (black and white outer) surrounding a blank region in the centre that is coated with colloidal quantum dots (CQDs) in a polymer. (**b**) and (**c**) false-colour images showing the exponent extracted from a power fit on the emission-excitation curved measured across the area of a ‘fake’ tag (b) and a ‘real’ tag (**c**). The exponent in the centre of the QD region is lower degree than the surroundings. (**d**) For a series of ‘real’ (R1-5, containing CQDs in the centre region) and ‘fake’ (F1-3, no CQDs) tags, the difference in linearity is plotted, with whiskers on the boxes spanning two standard deviations.

To demonstrate the potential of using a non-linearity check for authenticating an optical tag based on QDs in the real world, a simple fabrication and measurement procedure are proposed in which a blank region in the centre of a QR Code is coated with CQDs embedded in lacquer, as shown in Fig. 4a. The process used to create these tags is detailed in the supplemental information §1. Emission from the centre region of these tags can be collected using a smartphone’s camera module, whilst varying the excitation intensity by controlling the strength at which the flash on the phone is fired. Blank regions from the tag, not containing QDs can be used to calibrate the strength of the flash and the response of the camera. For simplicity, for these smartphone measurements, no external filters or light sources were used.

Figure 4b shows an example of the results obtained using a smartphone to read these tags. Using the response of the tags at different illumination intensities, we can measure the linearity of the response of the quantum dots. Like with the microscope-measured samples, we observe a nonlinearity of emission with a linear increase in excitation energy. The deposition method creates a much more uniform distribution than the electrospray, so the change in linearity has a weaker spatial dependence, but it is still possible to characterise the nonlinearity with a smartphone. To quantify this nonlinearity, a power fit was performed on the emission intensity as a function of the flash strength, and from this, the power exponent was extracted. The nonlinearity coefficient is extracted from the expression of the form:

$$y= k{x}^{n}$$

where we define *n* as the nonlinear coefficient, describing the response of the QDs, as a function of incident power. When the samples are illuminated with a known incident power *x*, we obtain *n* from the response of the sample on the detector, where *y* is the response intensity of the sample. We expect a linear response for any region of samples without quantum dots, so the expected value of *n* is 1, which we used to scale all other responses from the QDs. Figure 4c shows a false-colour image illustrating the extracted coefficient for averaged regions across the tag.

For white areas of the tag containing no QDs, in the area surrounding the QR Code, for example, a linear response would be expected that would correspond to an exponent of 1. However, as a result of the calibration of the flash and response of the detector on the smartphone, the power index from the raw response appeared to be a super-linear response. Although the flash and detector calibration are unknown, the absolute value of the power index is unimportant, because we determine the relative linearity to the background pixels of the QR code. Because the response from the background regions would be expected to uniformly correspond to an exponent of 1, any variation in this optical response from the white background regions is attributed to being noise-dominated. Therefore, the background areas of the tags were linearised, so the average power index across this region was equal to unity. This fixed parameter was used when determining the nonlinearity coefficient across the sample. In doing this, we negate any issues where the intensity of the flash or the response of the detector is nonlinear. This is shown in Fig. 4b, where the linearity coefficient for background regions of the tag average at 1, and the quantum dot area in the centre of the tag have a sublinear response.

To show that tags that are made and measured in this way can be distinguished from a ‘counterfeit’, i.e. one made without using CQDs, measurements from a series of genuine (made with CQDs) tags were compared to fake counterparts. The averaged emission linearity of the fake tag is shown in Fig. 4b. To determine the distinguishability of these samples, the percentage difference in the average exponent of the power fit between areas in the corner of the QR code (which never contain CQDs) and the centre of the tag (in which only genuine tags will have CQDs) were calculated. The results are shown in Fig. 4d, as a box plot to illustrate the standard deviation of 25 measurements of both real and fake tags. The fake tags do not contain any emitting quantum dots, so the individual measurements of emission linearity are dominated by the reflectivity of the tag and the response of the flash and detector. The real tags with emitting QDs create a much more uniform response in linearity, where the fake tags have a much broader distribution, dominated by noise. We quantify this by calculating the standard deviation in percentage difference in linearity to be 0.94 for the real tags, compared with 1.42 for the fake tags. This effect is primarily due to the relative incident power between the optical microscope and the smartphone flash, causing saturation from the white background areas on the QR tag.

## Discussion

The behaviour of the tags created with CQDs in this study adds a dimension of security for position based optical PUFs, by verifying the presence of quantum emitters. These tags retain the advantages of the security from the physical position of the quantum dots within them, as has been previously investigated, but the addition of measuring the linearity of the response, and spatially dependent switch-on behaviour adds an additional layer of security through several additional checks of the optical response. Where conventional nanoparticle PUFs rely on high-resolution measurements of individual particles for their security, this new technique introduces additional checks to verify the tag’s authenticity, without complex measurements. The introduction of this measurement reduces the additional hardware requirements for high-resolution optics, conventionally required by these PUFs.

Further enhancement of the security of these devices could utilise other attributes of CQDs, such as blinking. Blinking is a common phenomenon in CQDs, in which there is an irregular series of bright and dark states formed in the quantum dot^26,27^. It is thought to originate from the formation of long-lived dark excitons in the dots^28,29^. Observation of this blinking effect could be used as a supplementary check which could be measured when verifying the authenticity of the QD-PUFs. Additionally, improvements could be made with the smartphone characterisation using a combination of the techniques discussed, by measuring both the linearity of QDs and macroscopic properties of the spatial position of the dots. The encoding capacity of this type of O-PUF, whilst discussed extensively in the literature, is directly related to the resolution of the optical measurement: the individual QDs are far smaller than the resolution capable in far-field optical techniques^30,31^.

It is important to introduce some practical considerations for the implementations of these devices in real world applications. An important factor to consider is the stability and longevity of QD-based optical PUFs. For this type of device, it could include stabilising the QDs with ligands, or embedding them within a polymer film, to prevent damage^32,33^.

In this work, we have demonstrated a simple method of extracting more information from a quantum dot physical unclonable function, by obtaining the incident power-dependent optical behaviour of the emitters in devices. This increases the security of these tags because it is less feasible for a nefarious party hoping to a clone of a tag that both reproduces a unique fingerprint extracted from the pattern of emission of the quantum dots in each tag, and also their non-linear optical response^34^. Creating tags containing CQDs is facile, whilst reproducing a clone that mimics the response of a genuine tag is hard. Further demonstration of successful measurement and distinguishability of devices with and without quantum dots, based on measurements with a smartphone, further validates the usefulness of this extra dimension of security for position-dependent quantum dot security tags.

## Methods and materials

Colloidal InP/ZnS core/shell QDs were obtained from NN labs. These have a core diameter of 3.9 nm and shell thickness of 1 nm. Total diameter 5.0 ± 0.5 nm. They have an emission peak of 620 ± 15 nm.

To measure the optical properties of the InP/ZnS QD films, a simple photoluminescence (PL) system was used. The sample was illuminated with filtered white light through a 50 × objective lens. The incident power was controlled with a variable ND filter, which then passed through a 550 nm short-pass filter. Emitted and reflected light from the sample is then passed through a 600 nm long-pass filter, ensuring only light emitted from the QDs was measured by the CCD, because light reflected with a shorter wavelength than that of the long-pass filter’s cut-on will be absorbed by it. The variable ND filter allows incident power on the sample to be adjusted, and therefore to measure the response from the QDs at different intensities.

The smartphone tags were characterised with a rear-facing wide-angle lens built into the iPhone X and its associated flash. The resolution of the primary camera on iPhones has remained 12MP since 2015 (iPhone 6S), so this resolution is representative of most modern smartphones. A custom-developed application was used to capture images of the sample illuminated between 0 and 100% incident power, with 20% intervals. The images were divided into several ‘averaged squares’, where the intensity of response for pixels within this region was averaged.

## Supplementary Information

Supplementary Information.

## Data Availability

The datasets generated and/or analysed during the current study are available in the Lancaster University Research Data repository, 10.17635/lancaster/researchdata/561
