# Supplementary material for: Using intrinsic properties of quantum dots to provide additional security when uniquely identifying devices
Source: Sci Rep. 2022 Oct 8;12:16919. doi: 10.1038/s41598-022-20596-8 (PMC9547896; doi:10.1038/s41598-022-20596-8)
Supplement: Supplementary file 1 — Supplementary Information. [file 41598_2022_20596_MOESM1_ESM.docx]

**Supporting Information**

Using intrinsic properties of quantum dots to increase security when uniquely identifying devices

Matthew J. Fong*^1^, Christopher S. Woodhead^1^, Nema Abdelazim^1^, Daniel Abreu^2^, Angelo Lamantia^1,2^ Elliott Ball^1^, Kieran Longmate^1^, David Howarth^2^, Benjamin J. Robinson^1^, Phillip Speed^2^, Robert J. Young*^1^

^1^ Physics Department, Lancaster University, Bailrigg, LA1 4YB, UK.

^2^ Quantum Base Ltd, Physics Department, Lancaster University, Bailrigg, LA1 4YB, UK.

1. Fabrication of smartphone-readable tags

1: Fabrication of smartphone-readable tags

A quantum dot mixture was fabricated by immersing CIS colloidal QDs in a flat-bottomed vial in a commercial UV lacquer with a concentration of 100mg/ml. This was mixed using a SciQuip Pro40 Digital Laboratory mixer, with a custom-designed 3D-printed tip, at a constant speed of 1000rpm for 1 hour to create a dense and viscous mixture. The vial was immersed in an ice bath, to prevent damage to the QDs, caused by excess heating.

Once mixed, the ink was sampled, to test for the presence of any unmixed material, and to check the optical properties of the ink.


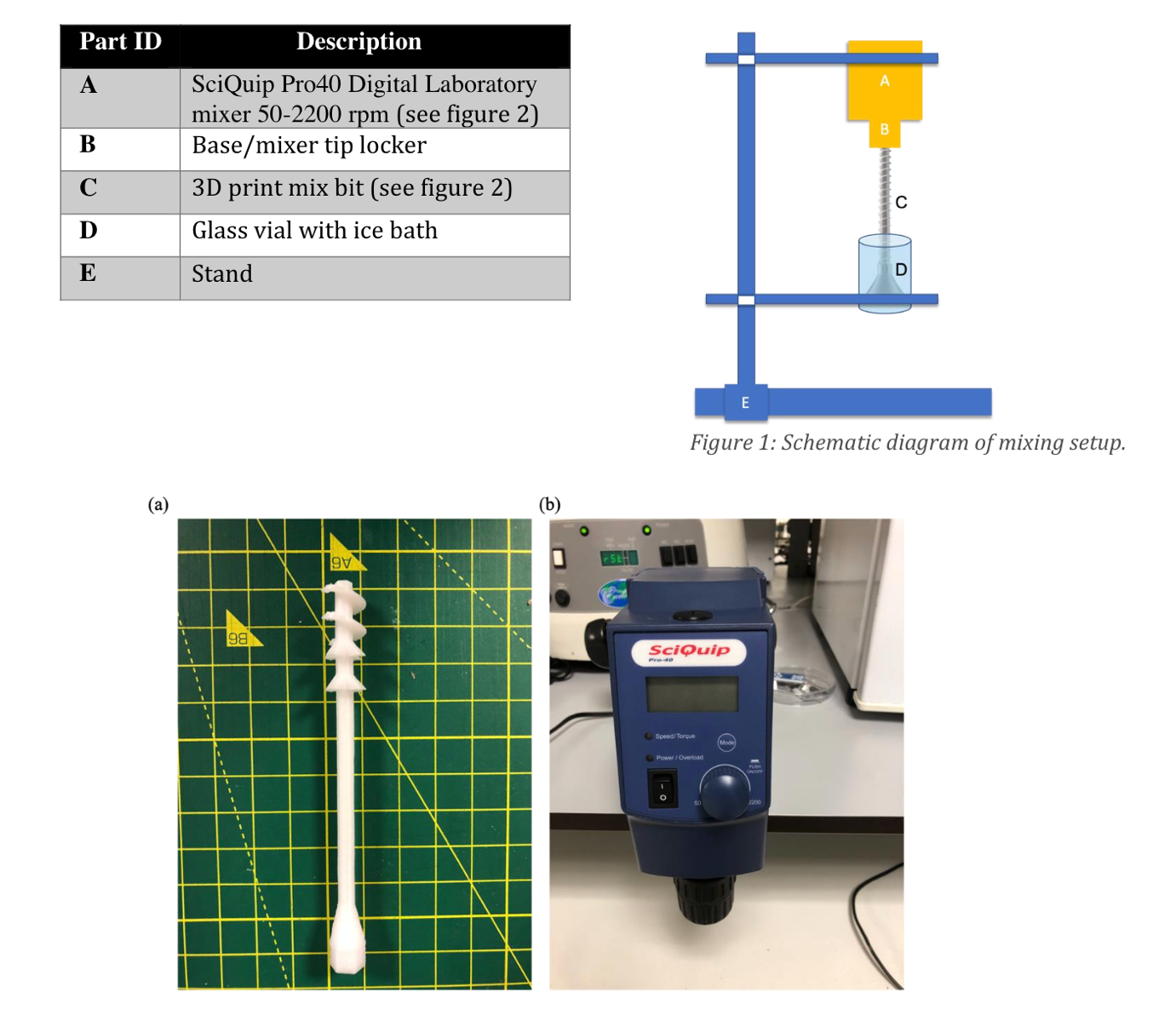


Figure S1: Schematic of mixing procedure for QD mixture


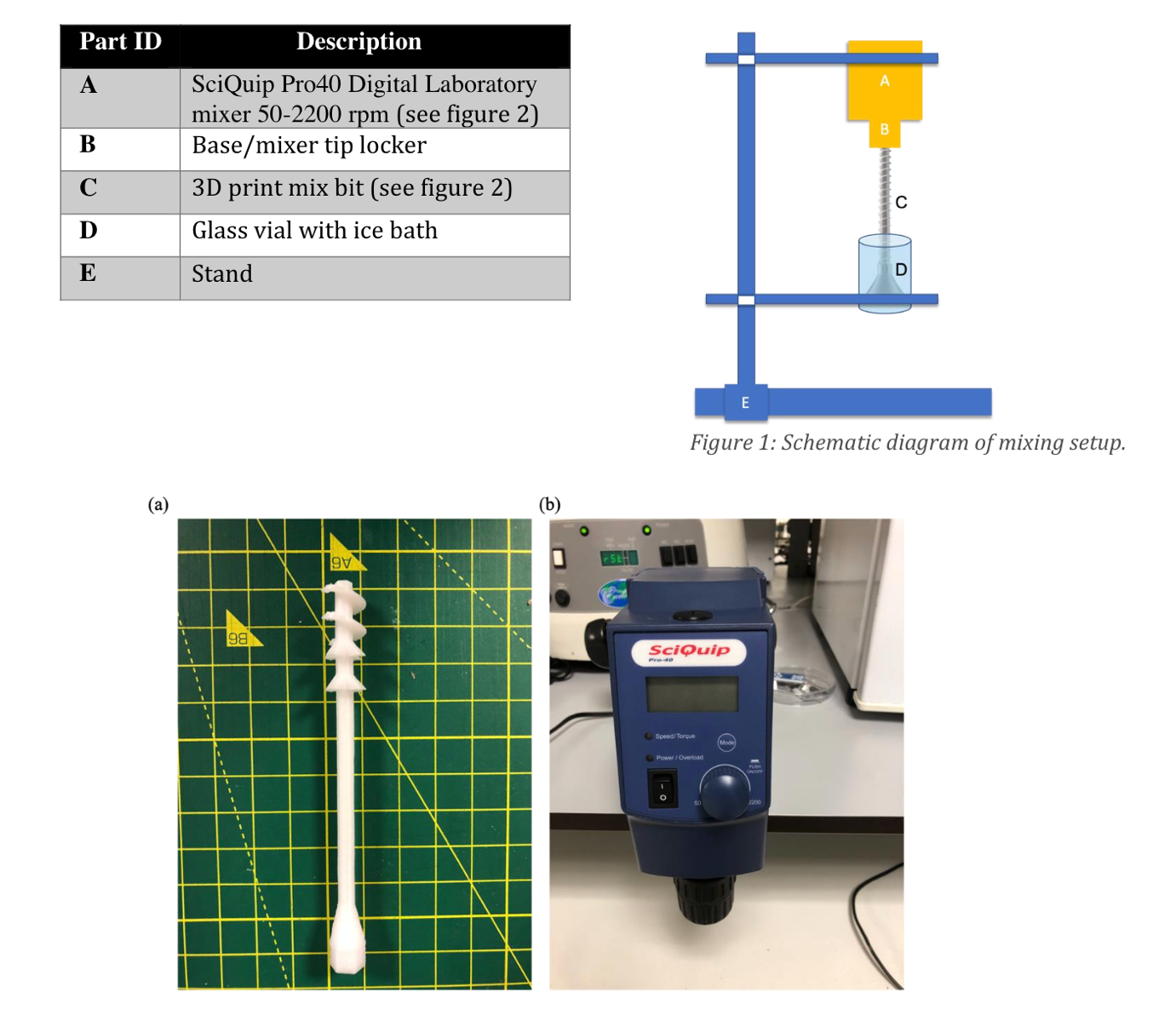


Figure S2: Custom 3D-printed stirrer for QD mixture

To produce the smartphone-readable tags, the QD ink was spread onto Raflatac POLYLASER substrate using an adjustable micro metric draw-down film applicator, forming a 2cm thin film of lacquer, with a thickness of 2 or 4μm. This film was then cured using a 1.5kW mercury lamp for 60s. This cured ink was then cut into 12mm squares and mounted at the centre of the QR code.
